# Alleviation of Cognitive Impairment-like Behaviors, Neuroinflammation, Colitis, and Gut Dysbiosis in 5xFAD Transgenic and Aged Mice by *Lactobacillus mucosae* and *Bifidobacterium longum*

**DOI:** 10.3390/nu15153381

**Published:** 2023-07-29

**Authors:** Xiaoyang Ma, Jeon-Kyung Kim, Yoon-Jung Shin, Young-Hoo Son, Dong-Yun Lee, Hee-Seo Park, Dong-Hyun Kim

**Affiliations:** 1 Neurobiota Research Center, College of Pharmacy, Kyung Hee University, Seoul 02447, Republic of Korea; 2 College of Pharmacy, Jeonbuk National University, Jeonju 54896, Republic of Korea

**Keywords:** cognitive decline, brain-derived neurotropic factor, *Lactobacillus mucosae*, *Bifidobacterium bifidum*, gut microbiota

## Abstract

Neuropsychiatric disorders including Alzheimer’s disease (AD) may cause gut inflammation and dysbiosis. Gut inflammation-suppressing probiotics alleviate neuropsychiatric disorders. Herein, to understand whether anti-inflammatory probiotics *Lactobacillus mucosae* NK41 and *Bifidobacterium longum* NK46, which suppressed tumor necrosis factor (TNF)-α expression in lipopolysaccharide (LPS)-stimulated macrophages, could alleviate cognitive impairment, we first examined their effects on cognitive function, gut inflammation, and gut microbiota composition in 5xFAD-transgenic mice. Oral administration of NK41 or NK46 decreased cognitive impairment-like behaviors, hippocampal amyloid-β (Aβ), TNF-α and interleukin (IL)-1β expression, hippocampal NF-κB^+^Iba1^+^ cell population, and Aβ accumulation, while hippocampal brain-derived neurotropic factor (BDNF) and IL-10 expression and BDNF^+^NeuN^+^ cell population increased. They also decreased TNF-α and IL-1β expression and NF-κB^+^CD11c^+^ cell population in the colon. They also reduced fecal and blood LPS levels and gut *Proteobacteria* and *Verrucomicrobia* populations (including *Akkkermansiaceae*), which are positively associated with hippocampal TNF-α and fecal LPS levels and negatively correlated with hippocampal BDNF level. However, they increased *Odoribactericeae*, which positively correlated with BDNF expression level and TNF-α to IL-10 expression ratio. The combination of NK41 and NK46 (4:1, NKc), which potently inhibited TNF-α expression in LPS-stimulated macrophages, additively alleviated cognitive impairment-like behaviors in 5xFAD-transgenic or aged mice. NKc increased hippocampal BDNF^+^NeuN^+^ cell population and BDNF expression in 5xFAD-transgenic or aged mice, while hippocampal TNF-α and IL-1β expression decreased. NKc also decreased TNF-α and IL-1β expression in the colon and LPS levels in the blood and feces. These findings suggest that gut bacteria and its product LPS may be closely connected with occurrence of cognitive impairment and neuroinflammation and the combination of NK41 and NK46 can additively alleviate cognitive impairment and neuroinflammation by inducing NF-κB-suppressed BDNF expression and suppressing LPS-producing gut bacteria.

## 1. Introduction

Alzheimer’s disease (AD) is a highly prevalent, neurodegenerative, and cognitive disorder in the elderly [1,2]. Aging is a main risk factor for the development of AD [3]. The pathological hallmark of AD patients is the accumulation of insoluble amyloid-β (Aβ) and hyperphosphorylated tau in the brain, which induce neuroinflammation and cortical and hippocampal cholinergic neurotransmission disruption [4,5]. Moreover, AD is occurred and deteriorated by microbial infection, gut microbiota dysbiosis, and their byproducts such as lipopolysaccharide (LPS) [6,7]. Anti-inflammatory probiotics alleviate AD in mice by the regulation of gut microbiota–gut–brain axis [8,9,10]. In the clinic study, anti-inflammatory therapy was suggested to be able to delay AD onset and progression [11,12,13].

Gut microbiota bidirectionally communicate with the brain through the microbiota-gut–brain axis [14]. Gut microbiota dysbiosis is closely associated with the occurrence of gastrointestinal inflammation and psychiatric disorders [15,16]. Gut microbiota dysbiosis-ameliorating probiotics have been reported to mitigate gastrointestinal inflammation and neuropsychiatric disorder including AD and depression [17,18]. *Lactobacillus plantarum* C29 improves cognitive decline and colitis in 5xFAD-transgenic mice [8]. *Lactobacillus mucosae* NK41 alleviates gut bacteria-induced memory impairment in mice by the attenuation of gut inflammation and dysbiosis [19]. *Bifidobacterium longum* NK46 also alleviates cognitive decline and colitis in 5xFAD-transgenic mice by the attenuation of gut dysbiosis [9]. The combination of *Lactobacillus acidophilus*, *Lactobacillus fermentum*, *Bifidobacterium lactis*, and *Bifidobacterium longum* alleviates Aβ-induced cognitive deficit in rats [20]. The combination of *Lactobacillus acidophilus*, *Lactobacillus casei*, *Bifidobacterium bifidum*, and *Lactobacillus fermentum* mitigates cognitive decline in AD patients [21]. These results support the suggestion that gut-dysbiosis-suppressing probiotics can simultaneously alleviate gastrointestinal inflammation and AD. Nevertheless, the action mechanism of probiotics against the cognitive impairment remain elusive.

In the preliminary study, NK41 and NK46 suppressed TNF-α expression in LPS-stimulated macrophages and improved LPS-impaired cognitive function in mice. Therefore, to confirm whether anti-inflammatory NK41 and NK46 could alleviate cognitive impairment, we examined the effects of NK41, NK46, and their combination (NKc) on cognitive function and gut microbiota composition in 5xFAD-transgenic mice. Furthermore, we investigated the effect of NKc on cognitive function and gut dysbiosis in aged mice.

## 2. Materials and Methods

### 2.1. Materials

A general anaerobic medium (GAM) and radioimmunoprecipitation assay (RIPA) lysis buffer were purchased from Nissui Pharm. Co., Ltd. (Tokyo, Japan) and Biosesang Inc. (Seongnam, Republic of Korea), respectively. Enzyme-linked immunosorbent assay (ELISA) kits for cytokines were purchased from Ebioscience (Atlanta, GA, USA). Antibodies for brain-derived neurotropic factor (BDNF) and NeuN were purchased from Millipore (Burlington, MA, USA). Antibody for NF-κB was purchased from Cell Signaling Technology (Beverly, MA, USA). Antibodies for Iba1 were purchased from Abcam (Cambridge, UK). 4′,6-Diamidino-2-phenylindole dilactate (DAPI) was purchased from Sigma (St. Louis, MO, USA). Enzyme-linked immunosorbent assay (ELISA) kits for TNF-α, IL-1β, and IL-10 were purchased from Ebioscience (Atlanta, GA, USA).

### 2.2. Animals

5xFAD-transgenic C57BL/6 mice (male, 4-month-old) were purchased from RaonBio Co., Ltd. (Yongin-Shi, Republic of Korea). Aged C57BL/6 mice (male, 18-month-old) were purchased from RaonBio Co., Ltd. Young C57BL/6 mice (male, 6-week-old) were purchased from Koatech Inc. (Seoul, Republic of Korea). Mice were fed with water and food ad libitum and were maintained in a controlled room (temperature, 22 °C ± 1 °C; humidity, 50% ± 10%; and light cycle, 12 h [07:00–19:00]). Mice were acclimatized for 7 days before being used in the experiment. All animal experiments were ethically approved by the Committee for the Care and Use of Laboratory Animals in the University (IACC, KHUASP(SE)-21-217) and were performed according to the Ethical Policies and Guidelines of Kyung Hee University for Laboratory Animals Care and Use.

**Figure 1:**
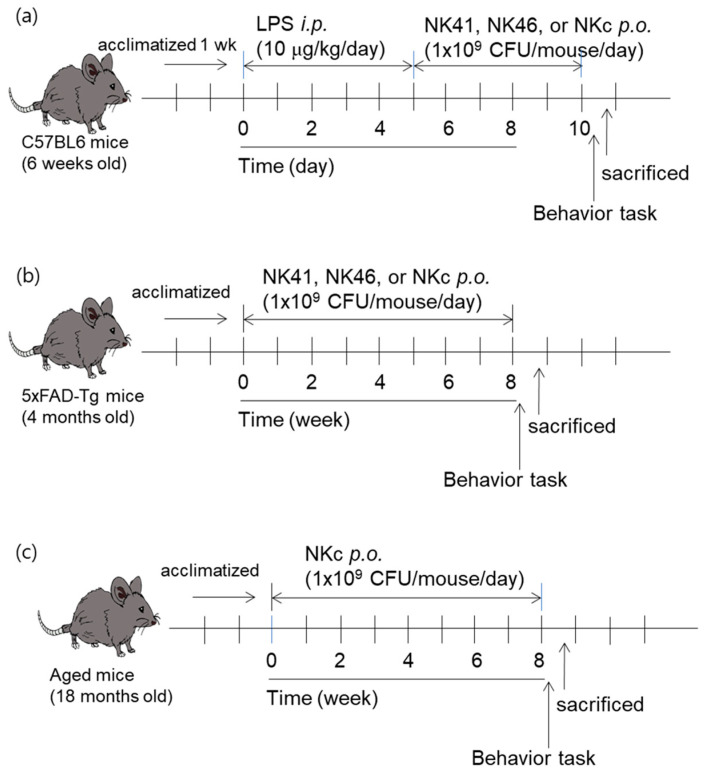
Experimental protocol in LPS-impaired mice (**a**), 5xFAD-transgenic mice (**b**), and aged mice (**c**).

First, we randomly divided mice in five groups (NC, Lp, LpL, LpB, and LpC). Each group consisted of 6 mice. Four groups (Lp, LpL, LpB, and LpC) were intraperitoneally injected with LPS (10 μg/kg/day) once a day for 5 days, as previously reported [22]. From the next day, test agents (Lp, vehicle; LpL, 1 × 10^9^ CFU/mouse/day of NK41; LpB, 1 × 10^9^ CFU/mouse/day of NK46; and LpC, 1 × 10^9^ CFU/mouse/day of NK41 and NK46 [4:1] mix [NKc], suspended in saline) were orally gavaged once a day for 5 days and their ameliorating effects on the cognitive decline were examined. NC group was treated with saline instead of test agents (Figure 1a).

**Figure 2:**
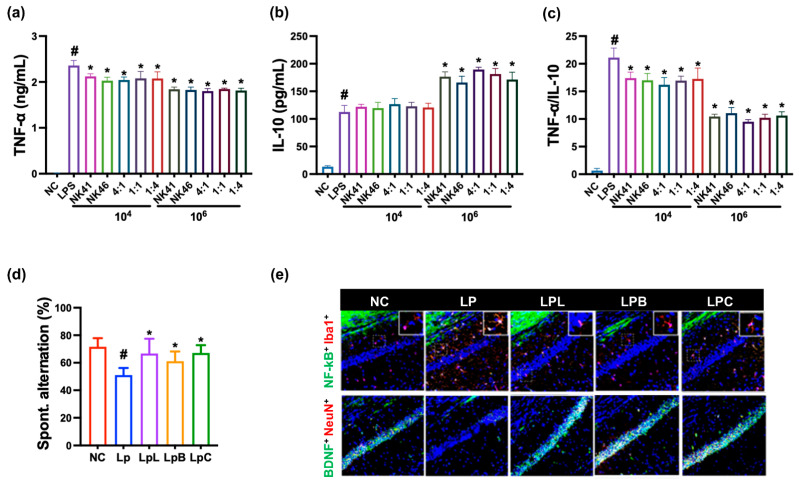
Anti-inflammatory effects of NK41, NK46, and NKc in LPS-stimulated macrophages and LPS-injected mice. Effects on TNF-α (**a**) and IL-10 expression (**b**) and their expression (TNF-α to IL-10) ratio (**c**) in LPS-stimulated macrophages. NK41, NK46, and NKc were treated with 1 × 10^4^ or 1 × 10^6^ CFU/mL). (**d**) Effects on spontaneous alternation in the Y-maze task. (**e**) Effects on the NF-κB^+^Iba1^+^ and BDNF^+^NeuN^+^ cell populations in the hippocampal CA1 region. Test agents (LpL, 1 × 10^9^ CFU/mice/day of NK41; LpB, 1 × 10^9^ CFU/mice/day of NK46; LpC, 110^9^ CFU/mice/day of NKc, NK41 and NK46 [4:1] mix) were orally administered. Normal control (Nc) and LPS-injected mice (Lp) were treated with vehicle instead of test agents. Data values were described as mean ± SD (*n* = 6). ^#^ *p* < 0.05 vs. Nc. * *p* < 0.05 vs. Lp.

Second, we randomly divided 5xFAD-transgenic mice in four groups (Tg, TgL, TgB, and TgC) and young healthy control mice in one group (Nc). Each group consisted of 6 mice. From the next day, test agents (Tg, vehicle; Tg41, 1 × 10^9^ CFU/mouse/day of NK41; Tg46, 1 × 10^9^ CFU/mouse/day of NK46; and TgM, 1 × 10^9^ CFU/mouse/day of NKc, suspended in saline) were orally gavaged once a day (6 times per week) for 8 weeks and their ameliorating effects on the cognitive decline were examined. Nc group was treated with saline instead of test agents (Figure 2b).

Third, we randomly divided aged mice in two groups (Ag and AgC) and young mice in one group (Yg). Each group consisted of 6 mice. From the next day, test agents [Ag, vehicle; and AgC, 1 × 10^9^ CFU/mouse/day of NK41 and NK46 (4:1) mix (NKc), suspended in saline] were orally gavaged once a day (6 times per week) for 8 weeks and examined their ameliorating effects on the cognitive decline. Yg group was treated with saline instead of test agents (Figure 1c).

Cognitive impairment-like behaviors were measured on the day after the final administration of test agents in the Y-maze, novel object recognition, and Barnes maze tasks. Mice were euthanized in a chamber filled with CO_2_, followed by cervical dislocation. Brains, colons, and bloods were collected for the determination of biomarkers.

For the immunofluorescence assay, mice were trans-cardiacally perfused with 4% paraformaldehyde. The hippocampus and colon tissues were collected, post-fixed with 4% paraformaldehyde, cryoprotected in 30% sucrose solution, frozen, and sectioned using a cryostat.

### 2.3. Behavioral Tasks

A Y-maze task was carried out in a three-arm (120°) horizontal maze (length, 40 cm; width, 3 cm; and wall height, 12 cm) illuminated around 150 lux, as previously reported [23]. A mouse was placed on an arm and the sequence and number of arm entries were recorded for 8 min. A spontaneous alternation was defined as entries into all three arms on consecutive choices. The ratio (%) of actual to possible alternations was calculated.

A novel object recognition test was performed in an open box (length, 45 cm; width, 45 cm; and height, 45 cm) made with a black acrylic panel, which was illuminated around 30 lux [24]. In the first training experiment, a mouse was placed in the box with two identical objects (cylinder: diameter, 3.4 cm; and height, 8 cm) a in the box, and freely exposed to the objects for 10 min. The second experiment was performed for 10 min 24 h after the first experiment. A mouse was placed in a box with one old object used in the first experiment and one new object (cuboid: length, 5 cm; width, 3 cm; and height, 8 cm) and recorded the number touching these objects for 10 min. Exploration (%) was indicated as the ratio percent of the sum of the new object-touching frequencies to the sum of all object-touching frequencies.

The Barnes maze task was performed in the maze consisted of a circular platform (diameter, 89 cm) with 20 holes (diameter, 5 cm) situated evenly around the perimeter and an escape box, which was located below the platform [25]. The room was illuminated around 250 lux. The training/acquisition phase was finished after a mouse entered the escape box. The test was maximally for 5 min. The mouse, which entered the box, was allowed to stay in the box for 30 s. If the mouse failed to enter the escape box within 5 min, it was led to the escape box. Mice were given two trials (for 5 min) each day for 5 consecutive days. The latency time to reach the escape hole was recorded.

### 2.4. Isolation and Culture of Macrophages

Macrophages were prepared, as reported previously [17]. Macrophage cells were then incubated with LPS (100 ng/mL, dissolved in saline) or vehicle in the absence or presence of probiotics (1 × 10^4^ or 1 × 10^6^ CFUs/mL) for 20 h. TNF-α and IL-10 levels were assayed using ELISA kits.

### 2.5. Immunoblotting and ELISA

Mouse brain and colon tissues were lysed with RIPA lysis buffer and centrifuged (10,000× *g*, 4 °C, 10 min). Cognition-related protein markers were measured by immunoblotting [26]. Levels of cytokines and myeloperoxidase in their supernatants were assayed by ELISA kits [26].

### 2.6. Immunohistochemical and Immunofluorescence Assay

For immunohistochemical assay, the sections of tissues (hippocampus) were washed with phosphate-buffered saline, blocked with serum, and immunostained with an antibody against Aβ42 (Invitrogen, Carbsband, CA, USA) to illuminate Aβ deposits, as previously reported [27]. For the immunofluorescence assay, the sections of tissues (hippocampus and colon) were washed with phosphate-buffered saline, blocked with serum, incubated with primary antibodies for Iba1 (1:200), NF-κB (1:100), LPS (1:150), BDNF (1:200), NeuN (1:200), Iba1 (1:200), and/or CD11c (1:150) overnight, and incubated with secondary antibodies conjugated with Alexa Fluor 488- or Alexa Fluor 594 (Invitrogen) for 1 h [25]. Cell nuclei were stained with DAPI. Immunostained sections were scanned with a confocal microscope.

### 2.7. Fecal Microbiota Analysis

Bacterial genomic DNA was isolated from the mouse fresh stool using a QIAamp DNA stool mini kit and amplified using barcoded primers (bacterial 16S rRNA V4 gene region) [19]. The amplicon sequencing was performed using Illumina iSeq 100 (San Diego, CA, USA). Sequenced reads were stored in the NCBI’s short read archive (accession number, PRJNA872311, https://www.ncbi.nlm.nih.gov/bioproject/PRJNA872311/ accessed on 12 June 2023).

### 2.8. Assays of Fecal and Blood LPS Levels

The contents of LPS in the blood and feces were assayed by using the diazo-coupled limulus amoebocyte lysate (LAL) assays, as previously reported [28]. For the blood LPS assay, bloods collected by retroorbital bleeding into ethylenediaminetetraacetic acid-coated BD Microtainer^®^ tubes (Becton Dickinson, Franklin Lakes, NJ, USA) were centrifuged at 13,000× *g* for 15 min. The supernatant (5 μL) was diluted 1:10 in pyrogen-free water and inactivated for 10 min at 70 °C. For the fecal LPS assay, feces were placed in 10 mL of phosphate-buffered saline, sonicated for 15 min on ice, and centrifuged at 400× *g* for 10 min. Supernatants were filtrated through a 0.45 μm Millipore filter, re-filtrated through a 0.22 μm filter, and inactivated at 70 °C for 10 min. LPS contents of filtrates and supernatants (50 μL) were assayed using the LAL assay kit according to the manufacturer’s protocol.

### 2.9. Whole Genome Analysis

The sequencing libraries were prepared according to the manufacturer’s instructions of 20-kb Template Preparation Using BluePippin™ Size-Selection System using PacBio DNA Template Prep Kit 1.0 [24]. NK41 genome sequence (1 contigs) were obtained by using PacBio RSII platform and NK46 genome sequence (2 contig) was completely obtained by using PacBio Sequel platform.

### 2.10. Statistical Analysis

Data are indicated as mean ± standard deviation (SD) and analyzed by Graph-Pad Prism 9. The significance was analyzed by Kruskal–Wallis test with Dunn’s post hoc test for non-parametric analysis (*p* < 0.05). The correlation between gut microbiota and spontaneous alternation, BDNF, TNF-α, or LPS level was analyzed using the Pearson correlation coefficient.

## 3. Results

### 3.1. Effects of NK41, NK46, and NKm on LPS-Induced Cognitive Impairment in Mice

First, to understand whether anti-inflammatory probiotics could alleviate cognitive impairment, we selected anti-inflammatory NK41 and NK46 using LPS-simulated macrophages and examined the effects of NK41, NK46, and NKc on LPS-induced cognitive impairment in mice (Figure 2). NK41 and NK46 strongly suppressed TNF-a expression and TNF-α to IL-10 expression ration in LPS-stimulated macrophages. When they were mixed, ([4:1], [1:1], and [1:4]), NKc [4:1] most potently suppressed TNF-α to IL-10 expression ratio. However, the anti-inflammatory effects between them were not significantly different. Oral administration of NK41, NK46, or NKc alleviated LPS-impaired cognitive function (spontaenous alternation) in the Y-maze task in mice. Furthermore, they also decreased LPS-induced activated microglia (NF-κB^+^Iba1^+^ cell) population in the hippocampus, while LPS-suppressed BDNF^+^NeuN^+^ cell population increased.

### 3.2. Effects of NK41, NK46, and NKc on Cognitive Function in 5xFAD-Transgenic Mice

**Figure 3:**
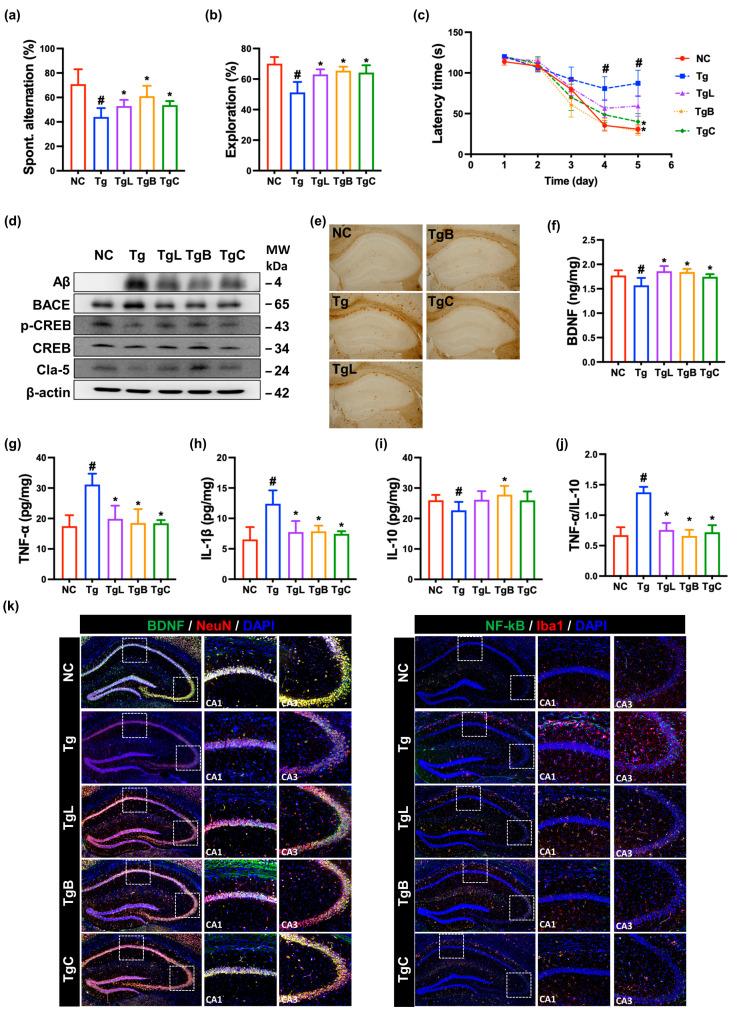
NK41, NK46, and NKc alleviated cognitive decline in 5xFAD transgenic (Tg) mice. (**a**) Effects on spontaneous alternation in Y-maze (**a**), novel object recognition (**b**), and Barnes maze tasks (**c**). (**d**) Effects on Aβ, BACE, claudin (Cla)-5, *p*-CREB, CREB, and β-action, assessed by immunoblotting. (**e**) Effect on the Aβ accumulation in the brain. Effects on BDNF (**f**), TNF-α (**g**), IL-1β (**h**), and IL-10 (**i**) expression and TNF-α to IL-10 expression ratio (**j**), assessed by ELISA. (**k**) Effects on BDNF^+^NeuN^+^ and NF-κB^+^Iba1^+^ cell populations in the hippocampus (Dotted squares CA1 and CA3 were expanded), assessed by immunofluorescence staining. (**j**) Test agents (Tg, vehicle; Tg41, 1 × 10^9^ CFU/mouse/day of NK41; Tg46, 1 × 10^9^ CFU/mouse/day of NK46; TgM, 1 × 10^9^ CFU/mouse/day of NKm) were orally administered. Normal control mice (NC) were treated with vehicle instead of test agents. Data were described as mean ± SD (*n* = 6). ^#^ *p* < 0.05 vs. Nc. * *p* < 0.05 vs. Tg.

Next, the effects of NK41, NK46, and NKc on the cognitive function were examined in 5xFAD-transgenic mice (Figure 3). The 5xFAD-transgenic mice exhibited the reduced spontaneous alternation in the Y-maze task to 62% [F(4,28) = 12, *p* < 0.001] of normal control mice and the reduced exploration in the novel object recognition test to 74% [F(4,28) = 15, *p* < 0.001] of control mice. Furthermore, although normal control mice significantly decreased the latency time to reach the escape hole for 5 consecutive days in the Barnes maze task, 5xFAD-transgenic mice did not significantly decrease it. The latency time of 5xFAD-transgenic mice increased to 266% [F(4,28) = 4, *p* = 0.009] of normal control mice. Moreover, amyloid-β (Aβ) and β-secretase (BACE) expression were significantly higher in the hippocampus of 5xFAD-transgenic mice (Tg) than in that of the control mice (Nc), while claudin-5 expression and cAMP response element-binding protein (CREB) phosphorylation were lower, and were assessed by immunoblotting (Figure 3d and Supplement Figure S1). Assessed by ELISA, TNF-α and IL-1β expression was higher in the hippocampus 5xFAD-transgenic mice, while BDNF and IL-10 expression was lower. Assessed by immunostaining, Aβ accumulation and NF-κB^+^Iba1^+^ cell population were significantly higher in the brain of 5XFAD-transgenic mice, while BDNF^+^NeuN^+^ cell population was lower. However, oral administration of NK41, NK46, or NKc (in 5xFAD-trangenic mice) increased spontaneous alteration in the YMT to 75%, 86%, and 76% [F(4,28) = 12, *p* < 0.001] of normal control mice (NC), respectively, and exploration in the novel object recognition test to 91%, 93%, and 92% [F(4,28) = 15, *p* < 0.001] of normal control mice (NC), respectively. Their treatments also reduced latency time in the BMT at the fifth day to 194%, 123%, and 115% [F(4,28) = 4, *p* = 0.009] of normal control mice (NC), respectively. Furthermore, treatment with NK41, NK46, or NKc decreased Aβ, BACE, Psen, TNF-α, and IL-1β expression, Aβ accumulation, NF-κB-positive cell numbers in the hippocampus and increased BDNF, claudin-5, and IL-10 expression, CREB phosphorylation, and BDNF^+^NeuN^+^ cell numbers.

### 3.3. Effects of NK41, NK46, and NKc on Gut Inflammation and Gut Microbiota Composition in 5xFAD-Transgenic Mice

**Figure 4:**
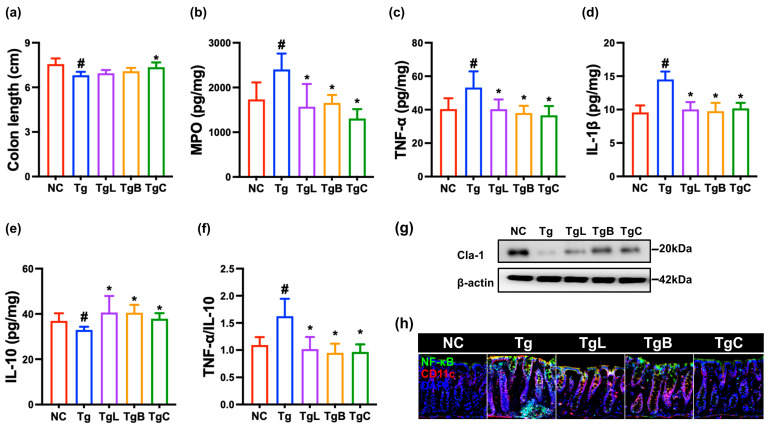
NK41, NK46, and NKc suppressed inflammatory marker levels in the colon of 5xFAD transgenic (Tg) mice. Effects on colon length (**a**), myeloperoxidase (MPO, (**b**)), TNF-α (**c**), IL-1β (**d**), and IL-10 expression (**e**), and TNF-α to IL-10 expression ratio (**f**). (**g**) Effects on the claudin (cla)-1 expression in the colon, assessed by immunoblotting. (**h**) Effects on NF-κB^+^CD11c^+^ cell population, assessed by immunofluorescence staining. Test agents (Tg, vehicle; TgL, 1 × 10^9^ CFU/mouse/day of NK41; TgB, 1 × 10^9^ CFU/mouse/day of NK46; TgC, 1 × 10^9^ CFU/mouse/day of NKc) were orally administered. Normal control mice (Nc) were treated with vehicle instead of test agents. Data were described as mean ± SD (*n* = 6). ^#^ *p* < 0.05 vs. Nc. * *p* < 0.05 vs. Tg.

We also investigated the effects of NK41, NK46, or NKc on inflammatory markers in the colon of 5xFAD-transgenic mice. Their treatments suppressed TNF-α, IL-1β, and myeloperoxidase expression and NF-κB^+^CD11c^+^ cell population and increased claudin-1 and IL-10 expression in the colon (Figure 4 and Supplement Figure S2).

**Figure 5:**
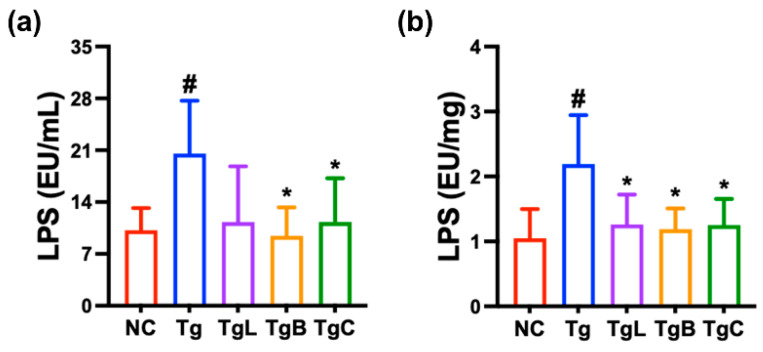
NKc decreased LPS level in the blood (**a**) and feces (**b**) of 5xFAD-transgenic mice (Tg). Test agents (Tg, vehicle; TgC, 1 × 10^9^ CFU/mouse/day of NKc) were orally administered. Healthy control (NC) were treated with vehicle instead of test agents. Data were described as mean ± SD (*n* = 6). ^#^ *p* < 0.05 vs. Nc. * *p* < 0.05 vs. Tg.

Next, to understand whether the gut microbiota endotoxin could be involved in the occurrence of systemic inflammation, we measured LPS levels in the blood and feces (Figure 5). The LPS level in the blood and feces was significantly higher in 5xFAD-transgenic mice than in healthy control mice. However, oral administration of NK41, NK46, or NKc significantly reduced LPS level in the blood and feces.

**Figure 6:**
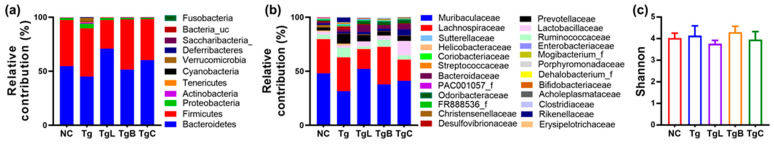

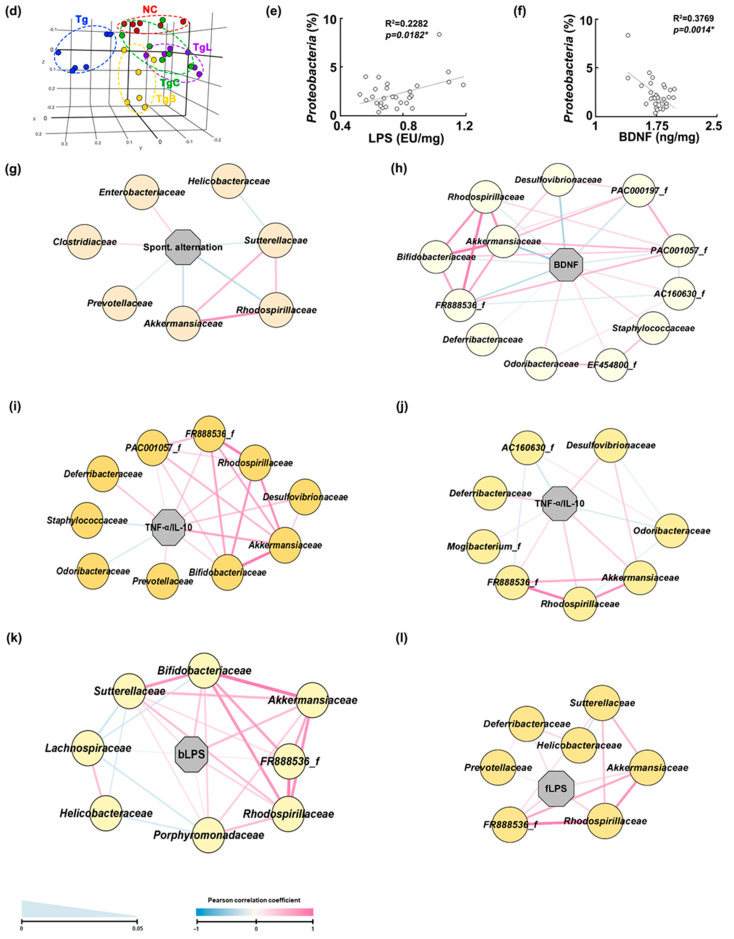
NK41, NK46, and NKc shifted gut microbiota composition in 5xFAD transgenic (Tg) mice. Effects on phylum (**a**) and family levels (**b**). Effects on α-diversity ((**c**), Shannon index) and β-diversity ((**d**), principal coordinate analysis plot based on Bray–Curtis analysis). The relationship between gut Proteobacteria composition and fecal LPS level (**e**) or hippocampal BDNF level (**f**), assessed by Pearson correlation analysis. The relationship between gut microbiota and spontaneous alternation (**g**), hippocampal BDNF (**h**), hippocampal TNF-α to IL-10 expression ratio (**i**), colonic TNF-α to IL-10 expression ratio (**j**), blood LPS (bLPS, (**k**)), or fecal LPS (fLPS, (**l**)), assessed by Pearson network analysis. Test agents (Tg, vehicle; TgL, 1 × 10^9^ CFU/mouse/day of NK41; TgB, 1 × 10^9^ CFU/mouse/day of NK46; TgC, 1 × 10^9^ CFU/mouse/day of NKc) were orally administered. Normal control mice (NC) were treated with vehicle instead of test agents. Data were described as mean ± SD (*n* = 6). * *p* < 0.05 vs. Tg.

We also investigated the effects of NK41, NK46, and NKc on the gut microbiota composition in 5xFAD-transgenic mice (Figure 6 and Supplement Tables S1 and S2). The gut microbiota composition and β-diversity of 5xFAD-transgenic mice were significantly different to that of healthy control mice. However, α-diversity was not different. It was found that 5xFAD-transgenic mice exhibited the higher abundance of Proteobacteria, Verrucomicrobia, Cyanobacteria, and Deferibactereres (including Ruminococcaceae, Prevotellaceae, Helicobacteriaceae, and Scutterellaceae) populations and the lower abundance of Bacteroidetes (including Muribaculaceae, Lactobacillaceae, and Odoribacteriaceae) population compared to healthy control mice. However, oral administration of NK41, NK46, or NKc partially shifted the β-diversity in 5xFAD mice to that in healthy control mice, while the α-diversity was not affected. They increased the population of Bacteroidetes including Muribaculaceae and Odoribacteriaceae and decreased Proteobacteria and Verrucomicrobia populations including Ruminococcaceae, Erysipelotrichacea Prevotellaceae, Helicobacteriaceae, and Scutterellaceae. At the phylum level, Proteobacteria population was positively correlated with fecal LPS level, while the hippocampal BDNF expression level was negatively correlated. At the family level, Rhodospiraceae, Akkermaniaceae, and Scutterllaceae populations were negatively correlated with spontaneous alternation, while Clostridiaceae and Enterobacteriaceae population were positively correlated. Odoribacteriaceae and AC160630_f populations were positively correlated with hippocampal BDNF level, while Akkermansiaceae, Desulfovibrionaceae, and FR888536_f populations were negatively correlated. Blood LPS level was positively correlated with Akkermanisaceae, Bifidobacteriaceae, and Rhodospirillaceae populations, while Lachnospiraceae population was negatively correlated. Fecal LPS level was positively correlated with FR888536_f, Rhodospirillaceae, Akkermansiaceae, Deferribacteraceae populations. Hippocampal and colonic TNF-α to IL-10 expression ratios were positively correlated with Akkermansiaceae, Deferribacteriaceae, Desulfovibrionaceae, and Rhodospirillaceae populations, while Odoribacteriaceae population was negatively correlated.

### 3.4. Effect of NKc on Cognitive Function in Aged Mice

**Figure 7:**
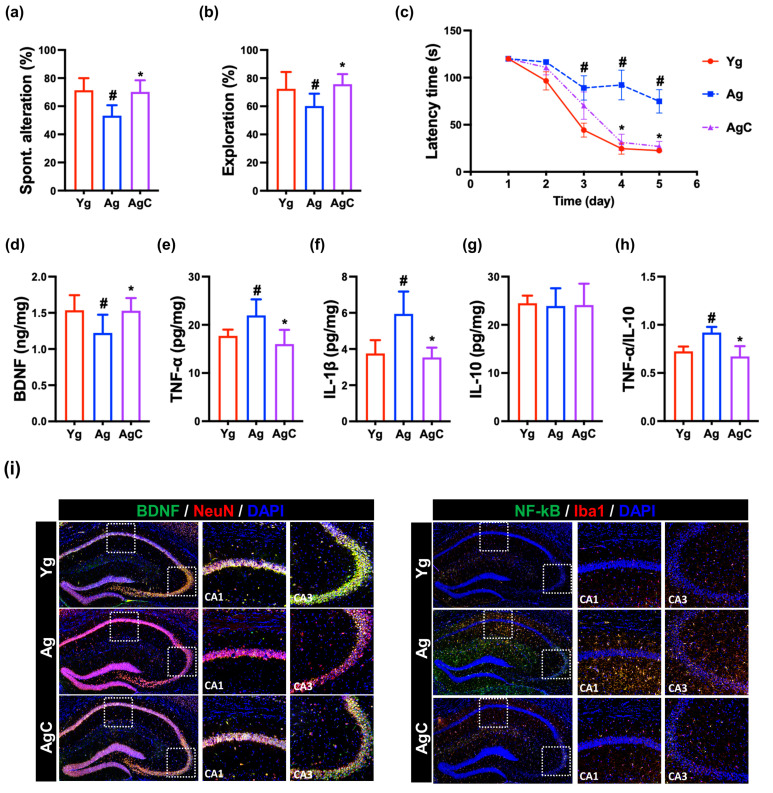
NK41 alleviated cognitive decline in aged mice (Ag). (**a**) Effects on spontaneous alternation in Y-maze (**a**), novel object recognition (**b**), and Barnes maze tasks (**c**). Effects on BDNF (**d**), TNF-α (**e**), IL-1β (**f**), and IL-10 (**g**) expression and TNF-α to IL-10 expression ratio (**h**), assessed by ELISA. (**i**) Effects on BDNF^+^NeuN^+^ and NF-κB^+^Iba1^+^ cell populations in the hippocampus, assessed by immunofluorescence staining. Test agents (Ag, vehicle; and AgC, 1 × 10^9^ CFU/mouse/day of NKm [NK41 and NK46 [4:1] mix]) were orally administered. Young mice (Yg) were treated with vehicle instead of test agents. Data were described as mean ± SD (*n* = 6). ^#^ *p* < 0.05 vs. Yg. * *p* < 0.05 vs. Ag.

First, we examined the effect of NKc on the cognitive function in aged mice (Figure 7 and Supplement Figure S3). The aged mice exhibited reduced spontaneous alternation in the Y-maze task to 75% [F(3,28) = 9, *p* < 0.001] of young mice, reduced exploration in the novel object recognition test to 78% [F(3,28) = 7, *p* = 0.002] of young mice, and increased latency time in the Barnes maze task to 353% [F(3,28) = 8, *p* < 0.001] of young mice. The p16, TNF-α, and IL-1β expression and NF-κB^+^Iba1^+^ cell (activated microglia) population were higher in aged mice than in young mice, while BDNF, IL-10, and claudin-5 expression, CREB) phosphorylation, and BDNF^+^NeuN^+^ cell population were lower in aged mice. However, oral administration of NK41 in aged mice increased spontaneous alteration in the YMT and exploration in the novel object recognition test to 90% [F(3,28) = 9, *p* < 0.001] and 97% [F(3,28) = 7, *p* = 0.002] of young mice, respectively, and reduced the latency time in the Barnes maze task (BMT) to 152% [F(3,28) = 8, *p* < 0.001] of young mice. Furthermore, p16, TNF-α, and IL-1β expression and activated microglial (NF-κB^+^Iba1^+^) cell numbers were higher in the hippocampus of aged mice than in that of young mice, while BDNF, claudin-5, and IL-10 expression, CREB phosphorylation, and BDNF^+^NeuN^+^ cell numbers were lower.

However, NKc treatment alleviated cognitive function including spontaneous alteration, exploration, and latency time in aged mice. NKc decreased p16, TNF-α, and IL-1β expression, and activated microglial cell (NF-κB^+^Iba1^+^) numbers in the hippocampus, while BDNF, claudin-5, and IL-10 expression, CREB phosphorylation, and BDNF^+^NeuN^+^ cell population increased (Figure 7 and Supplement Figure S1).

### 3.5. Effect of NKc on Gut Inflammation in Aged Mice

**Figure 8:**
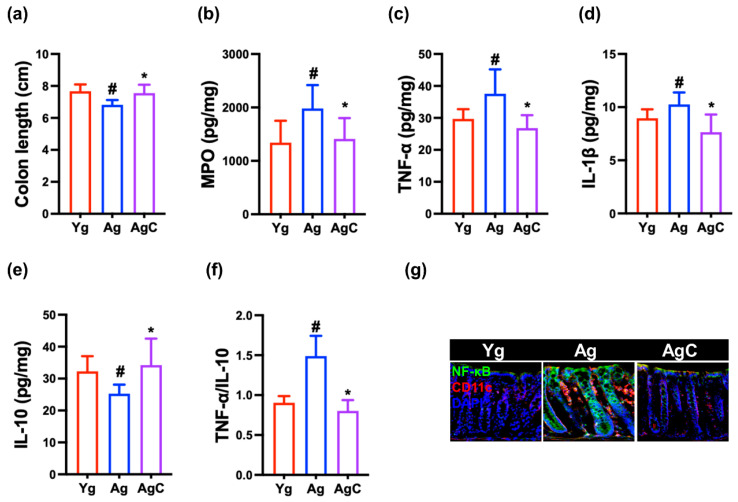
NKc decreased inflammatory marker levels in the colon of aged mice (Ag). Effects on colon length (**a**), myeloperoxidase (MPO, (**b**)), TNF-α (**c**), IL-1β (**d**), and IL-10 expression (**e**), and TNF-α to IL-10 expression ratio (**f**). (**g**) Effects on NF-κB^+^CD11c^+^ cell population assessed by immunofluorescence staining. Test agents (Ag, vehicle; AgC, 1 × 10^9^ CFU/mouse/day of NKc) were orally administered. Young mice (Yg) were treated with vehicle instead of test agents. Data were described as mean ± SD (*n* = 6). ^#^ *p* < 0.05 vs. Yg. * *p* < 0.05 vs. Ag.

Aged mice exhibited increased inflammatory marker (TNF-α and myeloperoxidase) levels in aged mice. However, oral administration of NKc decreased TNF-α, IL-1β, and myeloperoxidase expression and NF-κB^+^CD11c^+^ cell population and increased claudin-1 and IL-10 expression in the colon (Figure 8).

**Figure 9:**
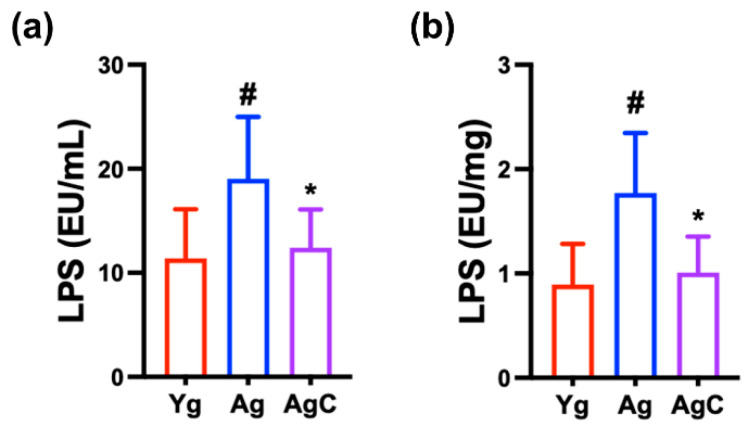
NKc decreased LPS level in the blood (**a**) and feces (**b**) of aged mice (Ag). Test agents (Ag, vehicle; AgC, 1 × 10^9^ CFU/mouse/day of NKc) were orally administered. Young mice (Yg) were treated with vehicle instead of test agents. Data were described as mean ± SD (*n* = 6). ^#^ *p* < 0.05 vs. Yg. * *p* < 0.05 vs. Ag.

Next, we also measured LPS levels in the blood and feces (Figure 9). The LPS level in the blood and feces was significantly higher in aged mice than in young control mice. However, oral administration of NKc significantly reduced LPS level in the blood and feces.

### 3.6. The Whole Genome Properties of NK41 and NK46

**Figure 10:**
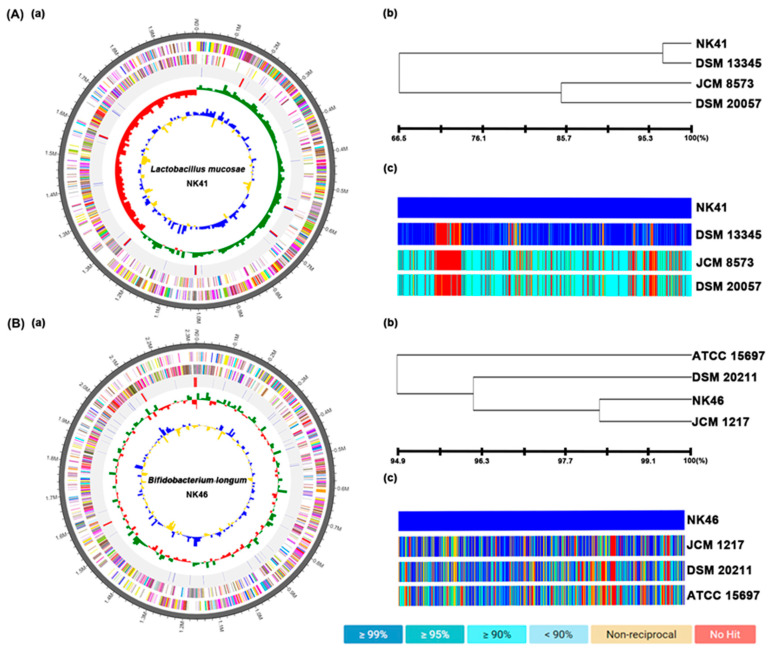
Taxonomic classification by genome-wide comparative analysis of NK41 (**A**) and NK46 (**B**). (**a**) The pseudochromosome drawn from 1 contig for NK41 and 2 contigs for NK46. The outermost circle means contig. The second inner circle is color-coded for the CDS information analyzed in the forward strand. The third inner circle is the CDS information analyzed in the reverse strand. The fourth circle from outside is tRNA (blue) and rRNA (red). The inner circle indicates GC skew metric information (green, higher than the average; red, lower than the average). The innermost circle is GC ratio metrics information (blue, higher values than average; yellow, lower values) GC skew and GC ratio metrics are displayed at 10 kb intervals. (**b**) Neighbor-joining tree based on the OrthoANI distance matrix (analyzed by UPGMA dendrogram, Newick format). (**c**) The pairwise ortholog matrix table (generated and colored according to the similarity between matching sequences).

To understand the genetic properties of NK41 and NK46, we analyzed their whole genome sequences (Figure 10). The genome of NK41 was 1,988,697 bp with a GC content of 46.7%. The total number of CDS was 1,843. The number of tRNA and rRNA genes were 98 and 21. The genome sequence of NK41 showed the phylogenetic similarity to Lactobacillus mucosae DSM13345 (96.72%), Lactobacillus buchneri subp. buchneri DSM20057 (66.7%), and Lactobacillus parakefiri JCM8573 (66.6%), using OrthoANI. The genome of NK46 was 2,513,457 bp with a GC content of 59.9%. The total number of CDS was 2,510. The number of tRNA and rRNA genes were 78 and 12. The genome sequence of NK46 showed the phylogenetic similarity to Bifidobacterium longum JCM1217 (98.4%), Bifidobacterium longum DSM20211 (96.2%), and Bifidobacterium longum ATCC15697 (94.6%), using OrthoANI.

## 4. Discussion

Aging-associated low-grade chronic inflammation as well as stress-induced gut inflammation and dysbiosis contribute to the development of AD [29,30]. In particular, Bifidobacteria and Lactobacilli populations were lower in the feces of elderly and aged mice than in young individuals and mice, respectively [31,32,33]. However, elderly and aged mice exhibited the higher Enterobacteriaceae population and bacterial LPS levels, which are closely connected with psychiatric disorders such as AD [32,34]. The gut microbiota of 5xFAD-transgenic mice, the well-known AD mouse model, also produce LPS more strongly than those of normal control mice [9]. In the present study, we also found that the LPS levels of 5xFAD-transgenic or aged mouse feces were higher than those of normal control mice. TNF-α and IL-1β expression and NF-κB-positive cell population were significantly higher in the hippocampus and colon of 5xFAD-transgenic or aged mice, while BDNF expression and BDNF-positive neural cell population were lower. In addition, Kim et al. reported that excessive expression of gut bacterial LPS caused gastrointestinal inflammation via the TLR4-linked NF-κB signaling [35]. Jang et al. reported that intraperitoneal injection of LPS caused cognitive impairment with neuroinflammation through the suppression of NF-κB activation-mediated BDNF expression [26]. Ma et al. reported that chronic exposure to gut microbiota dysbiosis-induced bacterial LPS could trigger the occurrence of neurodegenerative disorders such as AD [36]. Therefore, gut microbiota LPS is a risk factor for AD. Improving gut microbiota dysbiosis and suppressing gut bacterial LPS production may be beneficial for the therapy of cognitive decline in patients with AD and elderly.

In the present study, we found that NK41 and NK46 inhibited LPS-induced TNF-α expression in macrophages and NF-κB-positive cell population in mice. They alleviated LPS-induced cognitive impairment in the Y-maze test. They also suppressed TNF-α and IL-1β expression and NF-κB-positive cell population in the hippocampus and colon of 5xFAD-transgenic mice. They also suppressed Aβ and its related BACE expression and Aβ accumulation in the brain of 5xFAD-transgenic mice. However, they increased BDNF expression and BDBF^+^NeuN^+^ cell population in the hippocampus. Decourt et al. reported that TNF-α induced Aβ accumulation in the brain rodents [37]. Kim et al. reported that NK41 suppressed TNF-α expression and increased BDNF expression in the hippocampus of mice with Escherichia coli K1-induced cognitive function [19]. Lee et al. reported that NK46 suppressed TNF-α expression and increased BDNF expression in the hippocampus of 5xFAD-transgenic mice [9]. These results suggest that anti-inflammatory probiotics NK41 and NK46 may decrease TNF-α and Aβ expression through the suppression of NF-κB activation, resulting in the increase in BDNF expression and BDNF-positive cell population in the brain. We also found that NK41 suppressed TNF-α, IL-1β, and p16 expression and increased BDNF expression and BDNF-positive cell population in hippocampus of aged mice (Supplement Figure S1). Lee et al. reported that NK46 suppressed TNF-α, IL-1β, and p16 expression in the hippocampus of aged mice. These results suggest that NK41 and NK46 can suppress TNF-α and p16 expression through the inhibition of NF-κB activation, resulting in the increase in BDNF expression and BDNF-positive cell population in the brain. We also found that NK41 and NK46 alleviated cognitive impairment-like behaviors in 5xFAD-transgenic or aged mice. The NF-κB activation in immune cells stimulates inflammatory response in the brain and suppresses BDNF expression in neuron cells of brain [38,39]. BDNF induces long-term potentiation in neuron cells, leading to the increase in the cognitive function [40,41]. These results suggest that NK41 and NK46 may increase cognitive decline through the induction of NF-κB-suppressed BDNF expression.

NKc, the combination of NK41 with NK46, also suppressed TNF-α, IL-1β, and Aβ expression, Aβ accumulation, and NF-κB-positive cell population in the hippocampus of 5xFAD-trangenic mice. NKc also suppressed TNF-α, IL-1β, and p16 expression and NF-κB-positive cell population in the hippocampus of aged mice. NKc also suppressed TNF-α and IL-1β expression and NF-κB-positive cell population in the colon of 5xFAD-transgenic or aged mice. Although the effect of NKc was higher than that of NK41 or NK46 alone, it was not different between them. These results suggest that the combination of N41 with NK46 may additively alleviate cognitive impairment-like symptoms.

We also found that 5xFAD-transgenic mice exhibited a higher abundance of gut Proteobacteria and Verrucomicrobiota populations compared to control mice. Fecal and blood LPS levels were higher in 5XFAD-transgenic or aged mice than in control mice. Lee et al. reported that Proteobacteria population and LPS levels were significantly higher in aged mice than in young mice [9]. Yun et al. also reported that the gut microbiota of 5xFAD-transgenic mice strongly increased Proteobacteria population and LPS production [42]. However, we found that oral administration of NK41, NK46, and NKc reduced LPS levels in the blood and feces of 5xFAD-transgenic or aged mice. They reduced the population of Proteobacteria and Verrucomicrobia, which were positively correlated with the fecal or blood LPS level and negatively correlated with hippocampal BDNF expression level, in the gut microbiota of 5xFAD-transgenic mice. They also suppressed Akkermansiaceae, Sutterellaceae, and Desulfovibrionaceae populations, which were positively correlated with the fecal and blood LPS levels and hippocampal and fecal TNF-α to IL-10 expression ratios and negatively correlated with BDNF expression and spontaneous alternation. However, NK41, NK46, and NKc increased Odoribacteriaceae population, which was negatively correlated with fecal and blood LPS levels and hippocampal and fecal TNF-α to IL-10 expression ratios and positively correlated with BDNF expression and spontaneous alternation. These results suggest that NK41, NK46, and their mix NKc can alleviate systemic inflammation including neuroinflammation and colitis with gut dysbiosis by suppressing gut microbiota LPS production through the regulation of gut microbiota, resulting in the alleviation of cognitive impairment. Moreover, altough the genome sequences of NK41 and NK46 showed the phylogenetic similarity to Lactobacillus mucosae DSM13345 (96.7%) and Bifidobacterium longum JCM1217 (98.4%), respectively, their genome sequences were unique. Their cognitive-impairment-ameliorating effects may be due to their unique characteristics.

## 5. Conclusions

Probiotics NK41 and NK46 alleviated cognitive decline and neuroinflammation in 5xFAD-transgenic, aged, LPS-stimulated mice. NK41 and NK46 induced BDNF and NMDAR expression in the brain. They also alleviated colitis and gut microbiota dysbiosis. The combination of NK41 with NK46 additively alleviated cognitive decline, neuroinflammation, and colitis by up-regulation of BDNF signaling and down-regulation of NF-κB signaling and gut microbiota dysbiosis.

## Data Availability

The datasets used and/or analyzed during the current study are available from the corresponding author on reasonable request.

## Supplementary Materials

The following supporting information can be downloaded at: https://www.mdpi.com/article/10.3390/nu15153381/s1. Table S1. Effects of NK1, NK46, and NKc on the gut microbiota composition at the phylum level. Table S2. Effects of NK1, NK46, and NKc on the gut microbiota composition at the family level. Figure S1. Effect of NK41, NK46, and NKc on amyloid-β (Aβ), BACE, claudin (Cla)-5, *p*-CREB, CREB, caspase-3, and β-action expression in the hippocampus, assessed by immunoblotting. Figure S2. Effect of NK41, NK46, and NKc on claudin (Cla)-1 and β-action expression in the hippocampus, assessed by immunoblotting. Figure S3. Effects of NK41 and NKc on cognitive function in aged (Ag) mice.

## References

[1] Scheltens P., Blennow K., Breteler M.M., de Strooper B., Frisoni G.B., Salloway S., Van der Flier W.M. (2016). Alzheimer’s disease. Lancet.

[2] Cao Q., Tan C.C., Xu W., Hu H., Cao X.P., Dong Q., Tan L., Yu J.T. (2020). The Prevalence of Dementia: A Systematic Review and Meta-Analysis. J. Alzheimer’s Dis..

[3] Guerreiro R., Bras J. (2015). The age factor in Alzheimer’s disease. Genome Med..

[4] Ardura-Fabregat A., Boddeke E., Boza-Serrano A., Brioschi S., Castro-Gomez S., Ceyzériat K., Dansokho C., Dierkes T., Gelders G., Heneka M.T. (2017). Targeting Neuroinflammation to Treat Alzheimer’s Disease. CNS Drugs.

[5] Heneka M.T., Carson M.J., El Khoury J., Landreth G.E., Brosseron F., Feinstein D.L., Jacobs A.H., Wyss-Coray T., Vitorica J., Ransohoff R.M. (2015). Neuroinflammation in Alzheimer’s disease. Lancet Neurol..

[6] Fülöp T., Munawara U., Larbi A., Desroches M., Rodrigues S., Catanzaro M., Guidolin A., Khalil A., Bernier F., Barron A.E. (2020). Targeting Infectious Agents as a Therapeutic Strategy in Alzheimer’s Disease. CNS Drugs.

[7] Kim H.S., Kim S., Shin S.J., Park Y.H., Nam Y., Kim C.W., Lee K.W., Kim S.M., Jung I.D., Yang H.D. (2021). Gram-negative bacteria and their lipopolysaccharides in Alzheimer’s disease: Pathologic roles and therapeutic implications. Transl. Neurodegener..

[8] Lee H.J., Hwang Y.H., Kim D.H. (2021). Lactobacillus plantarum C29-Fermented Soybean (DW2009) Alleviates Memory Impairment in 5XFAD Transgenic Mice by Regulating Microglia Activation and Gut Microbiota Composition. Mol. Nutr. Food Res..

[9] Lee H.J., Lee K.E., Kim J.K., Kim D.H. (2019). Suppression of gut dysbiosis by Bifidobacterium longum alleviates cognitive decline in 5XFAD transgenic and aged mice. Sci. Rep..

[10] Galland L. (2014). The gut microbiome and the brain. J. Med. Food.

[11] Peila R., Launer L.J. (2006). Inflammation and dementia: Epidemiologic evidence. Acta Neurol. Scand. Suppl..

[12] Akiyama H., Barger S., Barnum S., Bradt B., Bauer J., Cole G.M., Cooper N.R., Eikelenboom P., Emmerling M., Fiebich B.L. (2000). Inflammation and Alzheimer’s disease. Neurobiol. Aging.

[13] Walker D., Lue L.F. (2007). Anti-inflammatory and immune therapy for Alzheimer’s disease: Current status and future directions. Curr. Neuropharmacol..

[14] Cryan J.F., O’Riordan K.J., Cowan C.S.M., Sandhu K.V., Bastiaanssen T.F.S., Boehme M., Codagnone M.G., Cussotto S., Fulling C., Golubeva A.V. (2019). The Microbiota-Gut-Brain Axis. Physiol. Rev..

[15] Parker A., Fonseca S., Carding S.R. (2020). Gut microbes and metabolites as modulators of blood-brain barrier integrity and brain health. Gut Microbes.

[16] Rogers G.B., Keating D.J., Young R.L., Wong M.L., Licinio J., Wesselingh S. (2016). From gut dysbiosis to altered brain function and mental illness: Mechanisms and pathways. Mol. Psychiatry.

[17] Yun S.W., Kim J.K., Lee K.E., Oh Y.J., Choi H.J., Han M.J., Kim D.H. (2020). A Probiotic Lactobacillus gasseri Alleviates Escherichia coli-Induced Cognitive Impairment and Depression in Mice by Regulating IL-1β Expression and Gut Microbiota. Nutrients.

[18] Guo L., Xu J., Du Y., Wu W., Nie W., Zhang D., Luo Y., Lu H., Lei M., Xiao S. (2021). Effects of gut microbiota and probiotics on Alzheimer’s disease. Transl. Neurosci..

[19] Kim J.K., Lee K.E., Lee S.A., Jang H.M., Kim D.H. (2020). Interplay between Human Gut Bacteria Escherichia coli and Lactobacillus mucosae in the Occurrence of Neuropsychiatric Disorders in Mice. Front. Immunol..

[20] Athari Nik Azm S., Djazayeri A., Safa M., Azami K., Ahmadvand B., Sabbaghziarani F., Sharifzadeh M., Vafa M. (2018). Lactobacilli and bifidobacteria ameliorate memory and learning deficits and oxidative stress in β-amyloid (1-42) injected rats. Appl. Physiol. Nutr. Metab..

[21] Akbari E., Asemi Z., Daneshvar Kakhaki R., Bahmani F., Kouchaki E., Tamtaji O.R., Hamidi G.A., Salami M. (2016). Effect of Probiotic Supplementation on Cognitive Function and Metabolic Status in Alzheimer’s Disease: A Randomized, Double-Blind and Controlled Trial. Front. Aging Neurosci..

[22] Lee D.Y., Kim J.K., Yun S.W., Han M.J., Kim D.H. (2021). DW2009 Elevates the Efficacy of Donepezil against Cognitive Impairment in Mice. Nutrients.

[23] Lee H.J., Lim S.M., Ko D.B., Jeong J.J., Hwang Y.H., Kim D.H. (2017). Soyasapogenol B and Genistein Attenuate Lipopolysaccharide-Induced Memory Impairment in Mice by the Modulation of NF-κB-Mediated BDNF Expression. J. Agric. Food Chem..

[24] Lee D.Y., Shin Y.J., Kim J.K., Jang H.M., Joo M.K., Kim D.H. (2021). Alleviation of cognitive impairment by gut microbiota lipopolysaccharide production-suppressing Lactobacillus plantarum and Bifidobacterium longum in mice. Food Funct..

[25] Lee K.E., Kim J.K., Han S.K., Lee D.Y., Lee H.J., Yim S.V., Kim D.H. (2020). The extracellular vesicle of gut microbial Paenalcaligenes hominis is a risk factor for vagus nerve-mediated cognitive impairment. Microbiome.

[26] Jang S.E., Lim S.M., Jeong J.J., Jang H.M., Lee H.J., Han M.J., Kim D.H. (2018). Gastrointestinal inflammation by gut microbiota disturbance induces memory impairment in mice. Mucosal Immunol..

[27] Korkmaz O.T., Ay H., Aytan N., Carreras I., Kowall N.W., Dedeoglu A., Tuncel N. (2019). Vasoactive Intestinal Peptide Decreases β-Amyloid Accumulation and Prevents Brain Atrophy in the 5xFAD Mouse Model of Alzheimer’s Disease. J. Mol. Neurosci..

[28] Jang H.M., Lee K.E., Kim D.H. (2019). The Preventive and Curative Effects of Lactobacillus reuteri NK33 and Bifidobacterium adolescentis NK98 on Immobilization Stress-Induced Anxiety/Depression and Colitis in Mice. Nutrients.

[29] Kim S., Jazwinski S.M. (2018). The Gut Microbiota and Healthy Aging: A Mini-Review. Gerontology.

[30] Induri S.N.R., Kansara P., Thomas S.C., Xu F., Saxena D., Li X. (2022). The Gut Microbiome, Metformin, and Aging. Annu. Rev. Pharmacol. Toxicol..

[31] Arboleya S., Watkins C., Stanton C., Ross R.P. (2016). Gut Bifidobacteria Populations in Human Health and Aging. Front. Microbiol..

[32] Kim K.A., Jeong J.J., Yoo S.Y., Kim D.H. (2016). Gut microbiota lipopolysaccharide accelerates inflamm-aging in mice. BMC Microbiol..

[33] Toward R., Montandon S., Walton G., Gibson G.R. (2012). Effect of prebiotics on the human gut microbiota of elderly persons. Gut Microbes.

[34] Jeong J.J., Kim K.A., Hwang Y.J., Han M.J., Kim D.H. (2016). Anti-inflammaging effects of Lactobacillus brevis OW38 in aged mice. Benef. Microbes.

[35] Kim K.A., Gu W., Lee I.A., Joh E.H., Kim D.H. (2012). High fat diet-induced gut microbiota exacerbates inflammation and obesity in mice via the TLR4 signaling pathway. PLoS ONE.

[36] Ma X., Shin Y.J., Jang H.M., Joo M.K., Yoo J.W., Kim D.H. (2021). Lactobacillus rhamnosus and Bifidobacterium longum alleviate colitis and cognitive impairment in mice by regulating IFN-γ to IL-10 and TNF-α to IL-10 expression ratios. Sci. Rep..

[37] Decourt B., Lahiri D.K., Sabbagh M.N. (2017). Targeting Tumor Necrosis Factor Alpha for Alzheimer’s Disease. Curr. Alzheimer Res..

[38] Abd El-Rahman S.S., Fayed H.M. (2022). Improved cognition impairment by activating cannabinoid receptor type 2: Modulating CREB/BDNF expression and impeding TLR-4/NFκBp65/M1 microglia signaling pathway in D-galactose-injected ovariectomized rats. PLoS ONE.

[39] Xu T., Liu J., Li X.R., Yu Y., Luo X., Zheng X., Cheng Y., Yu P.Q., Liu Y. (2021). The mTOR/NF-κB Pathway Mediates Neuroinflammation and Synaptic Plasticity in Diabetic Encephalopathy. Mol. Neurobiol..

[40] Ying S.W., Futter M., Rosenblum K., Webber M.J., Hunt S.P., Bliss T.V., Bramham C.R. (2002). Brain-derived neurotrophic factor induces long-term potentiation in intact adult hippocampus: Requirement for ERK activation coupled to CREB and upregulation of Arc synthesis. J. Neurosci..

[41] Messaoudi E., Ying S.W., Kanhema T., Croll S.D., Bramham C.R. (2002). Brain-derived neurotrophic factor triggers transcription-dependent, late phase long-term potentiation in vivo. J. Neurosci..

[42] Yun S.W., Park H.S., Shin Y.J., Ma X., Han M.J., Kim D.H. (2023). Lactobacillus gasseri NK109 and Its Supplement Alleviate Cognitive Impairment in Mice by Modulating NF-κB Activation, BDNF Expression, and Gut Microbiota Composition. Nutrients.

