# Supplementary material for: Alleviation of Cognitive Impairment-like Behaviors, Neuroinflammation, Colitis, and Gut Dysbiosis in 5xFAD Transgenic and Aged Mice by Lactobacillus mucosae and Bifidobacterium longum
Source: Nutrients. 2023 Jul 29;15(15):3381. doi: 10.3390/nu15153381 (PMC10421059; doi:10.3390/nu15153381)
Supplement: Supplementary file 1 [file nutrients-15-03381-s001.zip › nutrients-2473654-supplementary.pdf]

[Supplement]

Alleviation of cognitive compairment-like behaviors, neuroinflammation, colitis, and gut dysbiosis in 5xFAD transgenic and aged mice by *Lactobacillus mucosae* and *Bifidobacterium longum*

Table S1. Effects of NK1, NK46, and NKc on the gut microbiota composition at the phylum level

Taxon Name	Composition (%)[1]				
	NC	Tg	TgL	TgB	TgC
Bacteroidetes	53.2±19.5	48.5±15.1	67.5±10.4	48.9±13.9	54.5±21.2
Firmicutes	43.9±19.2	40.8±17.5	30.0±11.2	48.8±13.5	43.1±20.8
Proteobacteria	1.9±0.5	4.3±2.1*	1.9±0.9#	1.5±1.0#	1.6±1.3#
Actinobacteria	0.5±0.2	1.3±1.1	0.4±0.5	0.5±0.2	0.5±0.3
Tenericutes	0.3±0.3	0.5±0.4	0.2±0.2	0.2±0.2	0.2±0.2
Cyanobacteria	0.1±0.1	0.3±0.2*	0.0±0.0#	0.1±0.1#	0.1±0.1#
Verrucomicrobia	0.0±0.0	3.8±3.0*	0.0±0.0#	0.0±0.0#	0.0±0.0#
Deferribacteres	0.0±0.0	0.5±0.7	0.0±0.0	0.0±0.0	0.0±0.0
Saccharibacteria_TM7	0.0±0.0	0.0±0.0	0.0±0.0	0.0±0.0	0.0±0.0
Bacteria-uc	0.0±0.0	0.0±0.0	0.0±0.0	0.0±0.0	0.0±0.0
Fusobacteria	0.0±0.0	0.0±0.0	0.0±0.0	0.0±0.0	0.0±0.0

[1] Mean ± SD. #p<0.05 vs. Nc. *p<0.05 vs. Tg.

Table S2. Effects of NK1, NK46, and NKc on the gut microbiota composition at the family level

Taxon Name	Composition (%)[1]				
	NC	Tg	TgL	TgB	TgC
Muribaculaceae	47.1±17.9	34.8±11.7	48.6±12.5	35.3±12.5	37.7±15.7
Lachnospiraceae	30.5±16.0	27.9±18.0	21.5±10.2	36.9±13.0	26.6±18.6
Ruminococcaceae	4.5±1.6	8.4±2.4*	3.3±2.2#	6.5±1.9	4.0±2.7#
Lactobacillaceae	3.3±2.6	2.1±1.3	4.2±4.0	4.0±2.3	11.3±22.5
Erysipelotrichaceae	3.1±4.8	1.7±1.4	0.1±0.1#	0.1±0.1#	0.1±0.1#
Prevotellaceae	2.5±2.2	8.8±3.2*	6.5±6.2	3.1±3.1#	3.9±4.4
Christensenellaceae	2.0±3.0	0.4±0.1	0.6±0.4	0.5±0.3	0.7±0.4
Rikenellaceae	1.5±0.7	1.6±0.8	2.5±1.2	2.3±0.5	4.8±4.2
Bacteroidaceae	1.1±0.9	2.7±1.6	5.9±4.5	3.2±0.8	4.5±3.9
Desulfovibrionaceae	0.8±0.5	2.2±2.2	0.8±0.4	0.4±0.3	0.8±0.7
Odoribacteraceae	0.8±0.7	0.3±0.2	3.0±1.6#	3.5±2.1#	3.2±3.1
Helicobacteraceae	0.6±0.4	0.7±0.7	0.2±0.3	0.4±0.6	0.5±0.7
Coriobacteriaceae	0.4±.2	0.4±0.2	0.2±0.1	0.2±0.0	0.4±0.2
Sutterellaceae	0.4±0.4	1.1±0.9	0.9±0.8	0.5±0.4	0.2±0.3
Clostridiaceae	0.2±0.2	0.0±0.0*	0.0±0.1	0.0±0.0#	0.1±0.2
Acholeplasmataceae	0.2±0.3	0.3±0.2	0.2±0.2	0.1±0.1#	0.1±0.2

Dehalobacterium–f	0.2±0.1	0.2±0.1	0.1±0.1	0.2±0.1	0.1±0.1
Bifidobacteriaceae	0.1±.1	1.0±0.9	0.2±0.4	0.2±0.2	0.1±0.2
Porphyromonadaceae	0.1±0.1	0.3±0.3	0.2±0.2	0.2±0.1	0.1±0.1
Mogibacterium–f	0.1±0.1	0.1±0.0	0.0±0.0#	0.0±0.0#	0.1±0.0
Enterobacteriaceae	0.1±0.1	0.0±0.0	0.0±0.0	0.0±0.0	0.0±0.0
AC160630–f	0.1±0.0	0.0±0.0*	0.3±0.1#	0.2±0.2	0.2±0.2
FR888536–f	0.1±0.1	0.3±0.2*	0.0±0.0#	0.1±0.1#	0.1±0.1#
PAC001057–f	0.1±0.1	0.2±0.2	0.0±0.0	0.1±0.1	0.0±0.0#
Streptococcaceae	0.1±0.1	0.0±0.0	0.0±0.0	0.0±0.0	0.0±0.1

1) Mean ± SD. #p<0.05 vs. Nc. *p<0.05 vs. Tg.

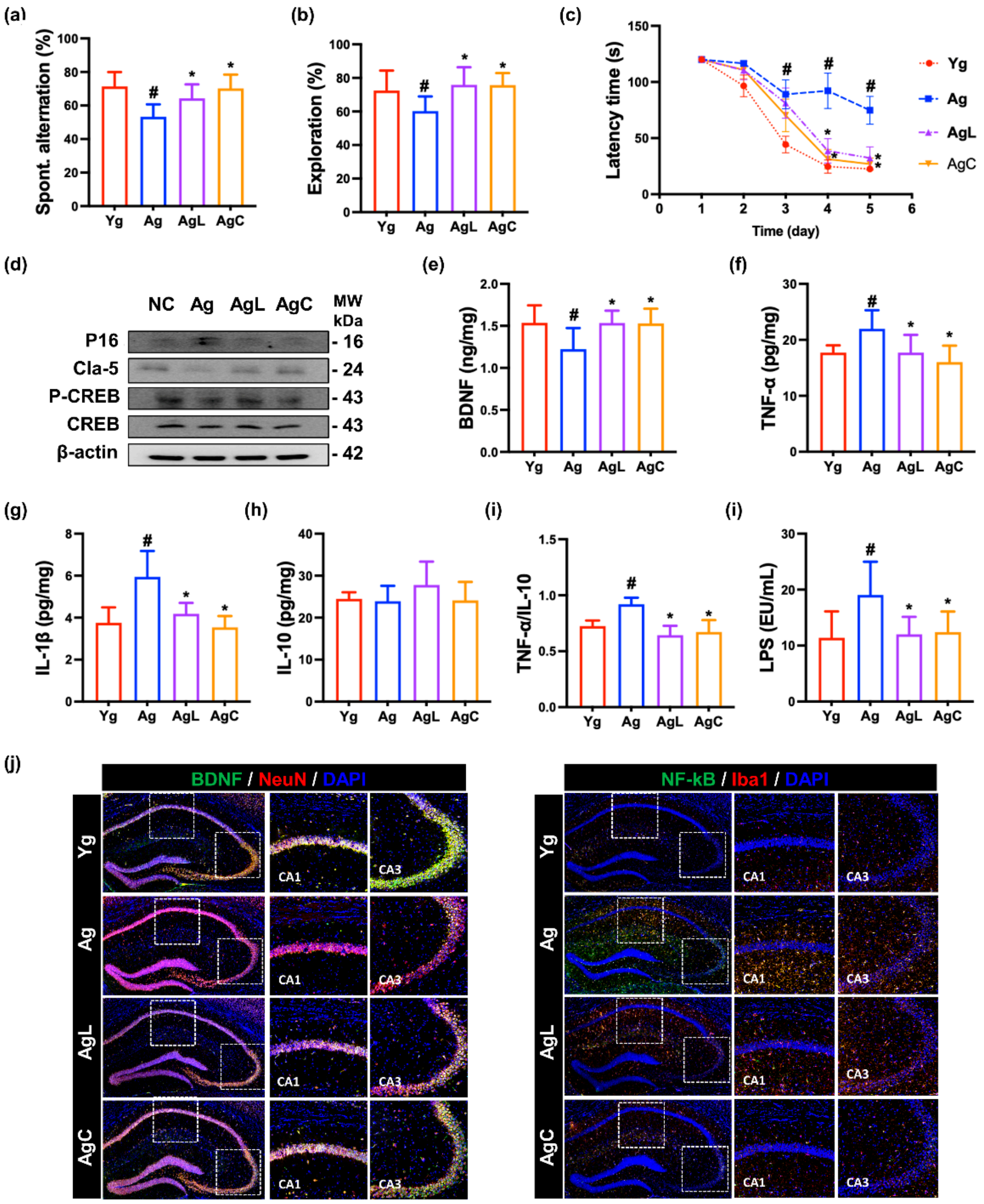

Figure S1. Effects of NK41 and NKc on cognitive function in aged (Ag) mice. Effects on spontaneous alternation in the Y-maze test (a), exploration in the novel object recognition test (b), and latency in Barnes maze task (c). (d) Effects on p16, claudin (Cla)-5, p-CREB, CREB, and β-action, assessed by immunoblotting. Effects on BDNF (e), TNF-α (f), IL-1β (g), and IL-10 (h) expression and TNF-α to IL-10 expression ratio (i), assessed by ELISA. (j) Effects on $BDNF^{+}NeuN^{+}$ and $NF\text{-}\kappa B^{+}Iba1^{+}$ cell populations in the hippocampus, assessed by immunostaining. Test agents (Ag, vehicle; Ag41, 1×10^{9} CFU/mouse/day of NK41; TgC, 1×10^{9} CFU/mouse/day of NKc) were orally administered. Young mice (Yg) were treated with vehicle instead of test agents. Data were described as mean ± SD (n = 6). $^{\#}p<0.05$ vs. Yg. $^{*}p<0.05$ vs. Ag.

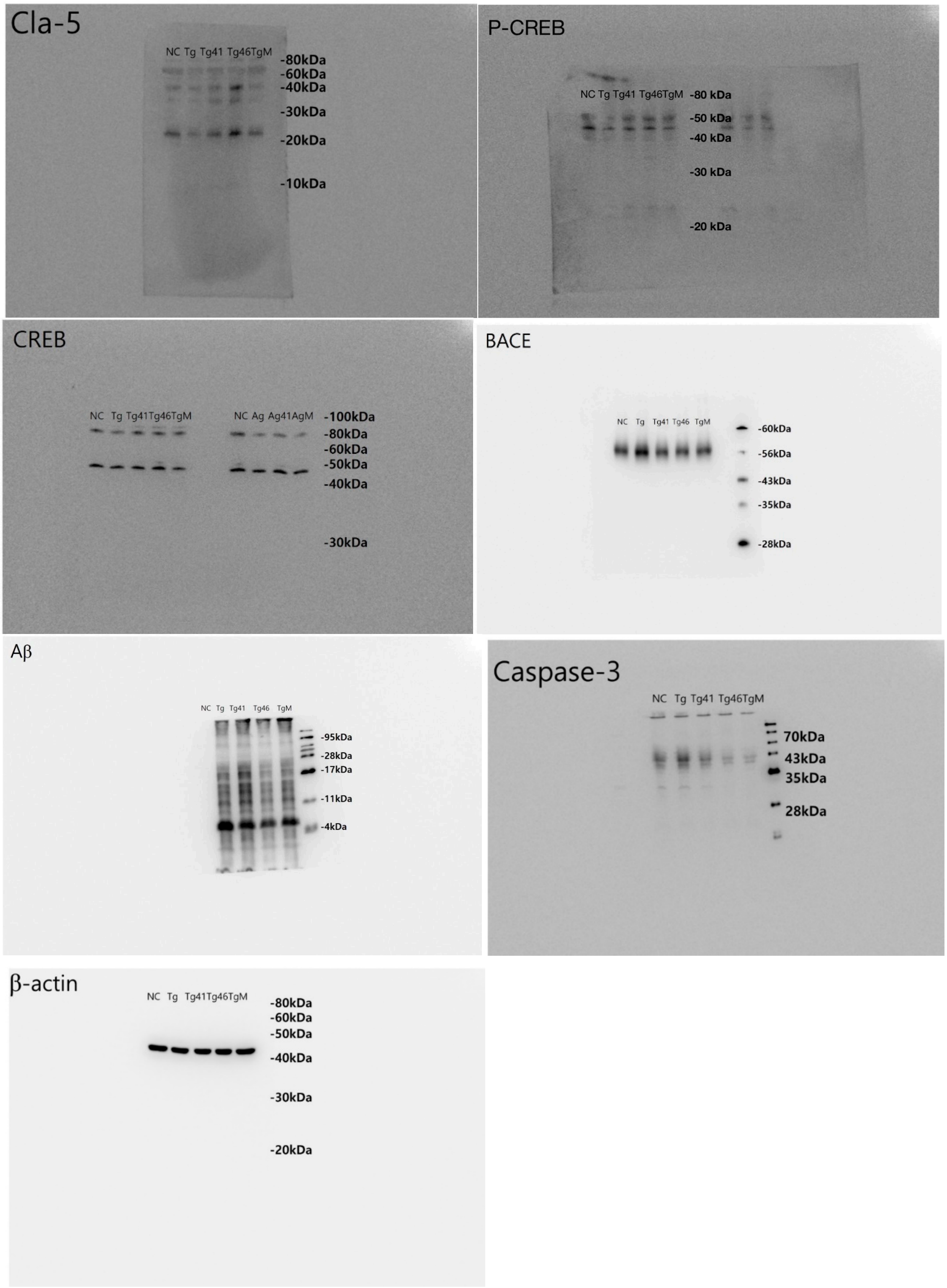

Figure S2. Effect of NK41, NK46, and NKc on amyloid-β (Aβ), BACE, claudin (Cla)-5, p-CREB, CREB, caspase-3, and β-action expression in the hippocampus, assessed by immunoblotting (in Figure 3d). Test agents (Tg, vehicle; Tg41 (TgL), 1×10^9 CFU/mice/day of NK41; Tg46 (TgB), 1×10^9 CFU/mice/day of NK46; TgM (TgC), 1×10^9 CFU/mice/day of NKm) were orally administered. Normal control mice (NC) were treated with vehicle instead of test agents.

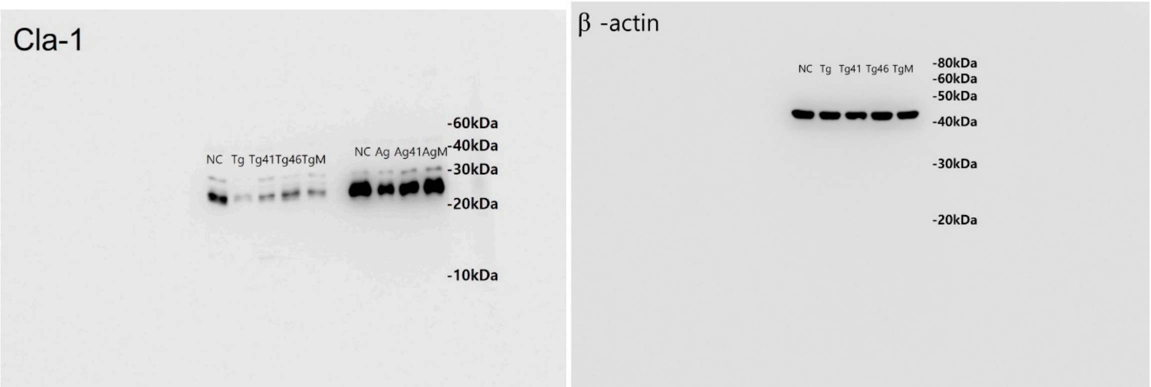

Figure S3. Effect of claudin (Cla)-1 and β-action expression in the hippocampus, assessed by immunoblotting (in Figure 4g). Test agents (Tg, vehicle; Tg41 (TgL), 1×10^9 CFU/mice/day of NK41; Tg46 (TgB), 1×10^9 CFU/mice/day of NK46; TgM (TgC), 1×10^9 CFU/mice/day of NKm) were orally administered. Normal control mice (NC) were treated with vehicle instead of test agents.
